# Application of panoramic radiographs in the diagnosis of temporomandibular disorders

**DOI:** 10.1097/MD.0000000000036469

**Published:** 2023-02-02

**Authors:** Xianbin Meng, Sen Liu, Zg Wu, Liangying Guo

**Affiliations:** a Department of Stomatology, The First Affiliated Hospital of Bengbu Medical College, Bengbu, China.

**Keywords:** panoramic radiographs, mandibular ramus height, condyle height, diagnosis, temporomandibular disorders

## Abstract

To evaluate the feasibility of temporomandibular disorder (TMD) diagnosis with panoramic radiography, and provide standardized data for artificial intelligence-assisted diagnosis by measuring the differences in the condylar and mandibular ramus heights. A total of 500 panoramic radiographs (219 male and 281 female participants) of healthy individuals were examined. The panoramic machine compatible measurement software, SCANORA 5.2.6, was used to measure the bilateral condylar height and mandibular ramus height, and SPSS 27.0 was used to calculate the left- and right-side differences in condylar height and mandibular ramus height of healthy individuals. Magnetic resonance images of the temporomandibular joint region obtained from 46 outpatients in the Stomatology Department were selected along with their corresponding panoramic radiographs. The left- and right-sided differences were measured and compared with the magnetic resonance imaging results. The measurement data are expressed as mean ± standard deviation (mm). *t* Tests were used to analyze data from healthy male and healthy female groups. The findings revealed that while there was no significant difference (*P* > .05) in the height of the condyle between men and women, there was a significant difference (*P* *<* .05) in the height of the mandibular ramus. In healthy population, the difference in height between the left and right condyle was 1.09 ± 0.99 mm. The difference in height of mandibular ramus in men was 1.26 ± 0.85 mm and that in women was 1.19 ± 0.87 mm. For the diagnosis of TMD, the sensitivity of panoramic radiographs was 94.74% (36/38), specificity was 75.00% (6/8), and diagnostic accuracy was 91.30% (42/46). The height of the right and left lateral condyles was not identical in healthy individuals, resulting in a discernible height discrepancy. In addition, the height of the mandibular ramus varied. By considering the left-right lateral height differences identified in this study along with clinical examination, it is possible to employ this metric as a preliminary screening tool for patients with TMD. Further, the use of panoramic radiographs for initial TMD screening is both viable and significant.

## 1. Introduction

Temporomandibular disorder (TMD) is a specific category of orofacial pain disorders. This category includes problems with the temporomandibular joint (TMJ), masticatory muscle exhaustion, jaw movement, and articular noise.^[1]^ The etiology of TMD is multifactorial. The most common factors are emotional stress, occlusal disturbances, tooth loss, postural deviations, masticatory muscle dysfunction, and structural changes in the internal and external TMJ. This increases the difficulty of early screening for TMD. The TMJ is the only bilateral linkage joint in the oral and maxillofacial region with a precise and complex structure, diverse functions, and a close relationship with occlusion. Owing to its complex anatomy and biomechanics, it is susceptible to pathological changes.^[2]^ The condyle is one of the most important structures in the TMJ and is closely related to the growth and development of the mandible. When the TMJ disc is displaced, the morphology of the condyle changes, leading to varying degrees of condylar resorption.^[3]^ The height of the condyle and mandibular ramus on the affected side significantly decreases. This modification results in a disparity in height between the condyles and mandibular ramus on the right and left sides, indicating the presence of TMD. Consequently, individuals with TMD can be distinguished by quantifying the dissimilarity between condylar and mandibular ramus measurements on the left and right sides.

Magnetic resonance imaging (MRI) can clearly show the morphology and position of the joint disc, as well as the state of the surrounding soft tissues, and therefore is considered the gold standard for the diagnosis of intra-articular TMD.^[4,5]^ According to 1 study, MRI is 96% reliable in identifying TMD.^[6]^ However, because of its high cost, and numerous contraindications,^[7,8]^ the clinical application of MRI is limited. If a patient has implanted metal devices, such as stents, pacemakers, steel nails, and plates, or has a fear of enclosed spaces (claustrophobia), MRI cannot be performed. Therefore, to overcome the shortcomings of MRI, it is particularly important to explore simple and rapid methods for diagnosing TMD. As the most commonly used auxiliary examination in stomatology, panoramic radiography has significant advantages in the study of jaw symmetry, owing to its economical, rapid, and unique imaging method.^[9]^ The benefits of panoramic radiography include customizable contrast, low radiation, quick imaging, and the ability to perform multi-angle observations. It has become a crucial auxiliary examination tool and is frequently used in the clinical diagnosis and treatment of stomatology. Although it is frequently employed in the diagnosis of conditions affecting the teeth and jaw, its significance in the diagnosis of condylar and mandibular symmetry has been disregarded.

The purpose of this study was to evaluate the feasibility of TMD diagnosis using panoramic radiography and to provide standardized data for artificial intelligence (AI)-assisted diagnosis by measuring the differences in the condylar and mandibular ramus height.

## 2. Materials and methods

### 2.1. Ethical considerations

Informed consent was obtained from all participants for this study. This study was approved by the Clinical Medical Research Ethics Committee of First Affiliated Hospital of Bengbu Medical College (approval number: 2023YJS199 and approval date: April 26, 2023).

### 2.2. Phase 1: Measurements of the condylar and mandibular ramus height

#### 2.2.1. Inclusion and exclusion criteria.

The inclusion criteria were as follows: the quality of the panoramic radiographs met the requirements of the National Radiology Department Quality Assurance, QC Academic Seminar Minutes; panoramic radiographs showed that the bilateral TMJ were normal and symmetrical; patients ranged in age of 20 to 45 years; and patients had no clinical signs of TMJ discomfort, such as joint pain, popping, or limited mouth opening. The exclusion criteria were as follows: deviation of the panoramic radiograph angle and positioning leading to obvious deformation of the image and uncooperative patient and refusal to consent to the panoramic imaging.

**Figure 1:**
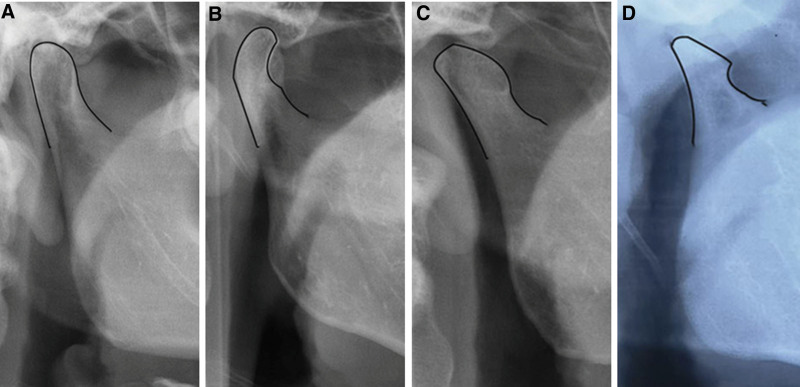
. The condyle morphology shown in panoramic X-ray: (A) Normal condyle morphology; (B) condyle narrow; (C) condylar anterior slope absorption; (D) idiopathic condylar resorption, ICR.

The various condylar morphologies shown in the panoramic radiographs are illustrated in Figure 1.

#### 2.2.2. Equipment and measurement methods.

##### 2.2.2.1. Equipment

The equipment used in this study was a panoramic machine (model number: Kavo Cranex D) from the Stomatology Department of the First Affiliated Hospital of Bengbu Medical College.

##### 2.2.2.2. Measurement methods

**Figure 2:**
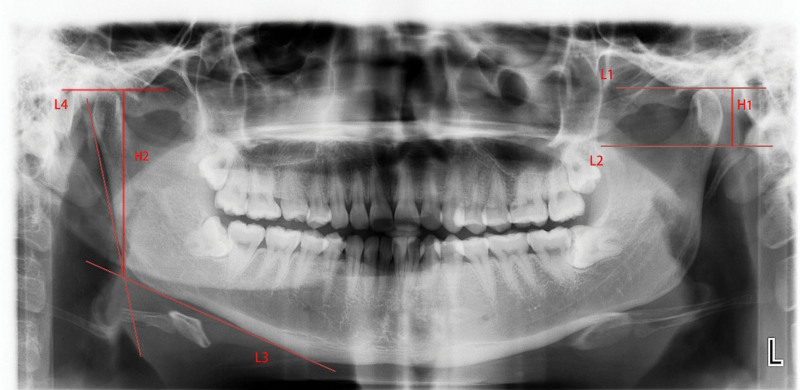
. Measurement schematic: L1: The horizontal line tangent to the highest point of the condyle; L2: The horizontal line tangent to the lowest point of sigmoid notch; L3: A line tangent to the most prominent point of the lower margin of the mandible ramus; L4: A line tangent to the posterior margin of mandibular ramus; H1: The vertical distance between L1 and L2; H2: The vertical distance between L3 and L4.

Condylar height was measured using 2 horizontal lines, L1 and L2, which are tangential to the highest point of the condyle and the lowest point of the sigmoid notch, respectively. The vertical distance between L1 and L2 was considered as the condylar height. Mandibular ramus height measurement was done using: tangent lines L3 and L4 through the inferior border of the mandibular ramus and the most prominent point of the posterior border of the mandibular ramus, respectively; horizontal line L1 tangent to the highest point of the condyle; and vertical distance from the intersection of L3 and L4 to L1 was calculated as the height of the mandibular ramus. The measurement schematic is shown in Figure 2.

To reduce the error caused by the measurement software and panoramic radiography, the above measurements were made using SCANORA 5.2.6, a measurement software that accompanies the panoramic machine Kavo Cranex D. Data were measured once by 2 senior attending physicians and 1 chief physician, and the average value was taken as the measurement.

### 2.3. Phase 2: MRI related

#### 2.3.1. Inclusion and exclusion criteria.

The inclusion criteria were as follows: in line with the requirements of the National Radiology Department Quality Assurance, QC Academic Seminar Minutes and patients age between 20 and 45 years. The exclusion criteria were as follows: the panoramic radiographs did not match the MRI findings and position of the joint disc was not accurate for various reasons.

#### 2.3.2. Equipment and diagnostic criteria for MRI.

##### 2.3.2.1. Equipment

The equipment used in this study was Philips MRI System (Model number: Achieva 3.0T) of the First Affiliated Hospital of Bengbu Medical College.

##### 2.3.2.2. Diagnostic criteria

In MRI recognition, the Wilkes classification^[10]^ was used as a criterion to determine TMJ status.

The panoramic films and MRI scans were reviewed for inclusion by 2 senior attending physicians and a chief physician, and the images in question were reviewed based on the chief physician assessment.

## 3. Results

### 3.1. Phase 1: Exploration of the difference values of condyle and mandibular rami

#### 3.1.1. General information.

According to the inclusion criteria, 500 healthy people panoramic radiographs were selected from the Department of Stomatology of the First Affiliated Hospital of Bengbu Medical College from January 2021 to December 2022, including 219 males and 281 females, aged from 20 to 45 years, with an average age of 33.27 ± 11.74 years.

#### 3.1.2. Test of normality.

Panoramic radiographs were divided into 3 groups: healthy males, healthy females, and healthy population (sum of males and females). The data were tested for normality and the results showed that all data conformed to a normal distribution (*P* *>* .05). Specific results are shown in Table 1.

**Table 1:** Normality test.

Group	Shapiro–Wilk			
	Left condyle height	Right condyle height	Left mandibular ramus height	Right mandibular ramus height
Healthy male	0.12	0.82	0.21	0.10
Healthy female	0.25	0.27	0.10	0.09
Healthy population	0.55	0.32	0.29	0.14

#### 3.1.3. Height measurement data.

All the datasets conformed to a normal distribution. The measurement data of the healthy male group, healthy female group, and healthy population group were expressed as mean ± standard deviation (mm). The results are shown in Table 2.

**Table 2:** height measurement data.

Group	Measurement data (mm)			
	Left condyle height	Right condyle height	Left mandibular ramus height	Right mandibular ramus height
Healthy male	20.15 ± 2.42	19.92 ± 2.25	64.75 ± 4.63	64.82 ± 4.69
Healthy female	20.04 ± 2.28	19.57 ± 2.30	60.16 ± 3.89	59.99 ± 3.87
Healthy population	20.09 ± 2.34	19.72 ± 2.28	61.41 ± 5.39	61.61 ± 5.06

#### 3.1.4. *t* Test results of male and female groups.

To determine whether there were sex differences in condylar and mandibular ramus heights, an independent-sample *t* test was used to analyze the data of the healthy male and healthy female groups. Statistical significance was set at *P* *<* .05, the data were statistically significant. The results are shown in Table 3. There was no significant difference in left condylar height between the healthy male and female groups, and right condylar height was also (*P* *=* .67 and *P* *=* .18). There was a significant difference in the height of the left mandibular ramus between the healthy male and healthy female groups, and the height of the right mandibular ramus was also (*P* *=* .00 and *P* *=* .00).

**Table 3:** *t* Test result.

Test index	Inspection item			
	Left condyle height	Right condyle height	Left mandibular ramus height	Right mandibular ramus height
*t* Value	0.41	1.33	9.52	9.84
*P* value	.67	.18	.00	.00

#### 3.1.5. Normal range of left-right difference.

SPSS 27.0 was used to calculate the 95% confidence interval of the left-right difference in condylar height in the healthy population group and the left-right difference in mandibular ramus height in the healthy male and female groups (Table 4).

**Table 4:** Left-right difference data.

Outcome index	The left-right difference		
	condyle height in healthy population	Mandibular ramus height in healthy male group	Mandibular ramus height in healthy female group
95% confidence interval	1.09 ± 0.99 mm	1.26 ± 0.85 mm	1.19 ± 0.87 mm

In summary, the height difference between the left and right condyle in a healthy population was 1.09 ± 0.99 mm. The difference in height of mandibular ramus in men was 1.26 ± 0.85 mm and that in women was 1.19 ± 0.87 mm.

### 3.2. Phase 2: Discussion on the feasibility of the diagnosis of TMD with panoramic radiographs

#### 3.2.1. General information.

According to the inclusion criteria, 46 patients’ MRI data and the corresponding panoramic radiographs were selected from the Department of Stomatology of the First Affiliated Hospital of Bengbu Medical College between January 2022 and January 2023, including 19 males and 27 females, aged from 20 to 45 years, with an average age of 32.34 ± 12.82 years.

#### 3.2.2. Test of diagnostic accuracy of panoramic radiographs.

In this study, 46 panoramic radiographs were measured. The left-right difference in condylar height and mandibular ramus height in 8 cases was within the normal range. Further, 38 cases indicated that the differences in mandibular ramus height, condylar height, or both were greater than the normal range. The maximum left-right difference of the condyle was 10.21 mm, and the minimum left-right difference was 3.61 mm. The median difference was 5.42 mm. The maximum left-right difference of the mandibular ramus was 12.15 mm, and the minimum difference was 2.83 mm. The median difference was 6.71 mm. The MRI data of the 46 patients were interpreted using the Wilkes classification. Among the 8 cases with normal left-right side difference, MRI revealed that 6 cases showed no obvious abnormality of the TMJ, 2 cases showed irreducible anterior displacement of articular disc; among the 38 cases with abnormal left-right disc difference, MRI revealed that 24 cases showed irreducible anterior displacement of the articular disc, 7 cases showed medial displacement of the articular disc, 5 cases showed irreducible anterior displacement of the articular disc with joint effusion, and 2 cases showed normal structure of joints bilaterally. The diagnostic results of MRI were used as the gold standard for TMD in this study, and the diagnostic results of MRI and panoramic radiographs were statistically analyzed. The results are shown in Table 5.

**Table 5:** Sensitivity and specificity.

Panoramic film	MRI		Total
	Abnormal	Normal	
Abnormal	36	2	38
Normal	2	6	8
Total	38	8	46

MRI = magnetic resonance imaging.

The results show that the sensitivity, specificity, and diagnostic accuracy of panoramic radiographs for the diagnosis of TMD were 94.74% (36/38), 75.00% (6/8), and 91.30% (42/46), respectively. The sensitivity was >90%, indicating that panoramic radiographs can be used for primary screening of TMD with a low rate of missed diagnoses. The specificity was <80%, suggesting that the misdiagnosis rate of panoramic radiographs for the primary screening of TMD needs to be minimized. Overall, the diagnostic accuracy was >90%, indicating that panoramic radiographs can be used for the initial clinical screening of TMD.

## 4. Discussion

### 4.1. Importance of TMJ research

The TMJ is one of the most active joints in the human body and has a strong tissue regeneration ability.^[11]^ The causes of TMD are extremely complex, and their incidence is increasing annually.^[12]^ It has been rated as the fourth oral epidemic by the World Health Organization.^[13]^ In recent years, it has become a research hotspot in the field of oral and maxillofacial surgery. Valesan et al found that the incidence in adults aged 20 to 30 years was 31% and the incidence in children and adolescents was approximately 11%.^[14]^ Alrashdan conducted a survey of a northern Jordanian population and found that the incidence of TMD was 26.7%.^[15]^ Silva conducted a meta-analysis of 11 surveys on the incidence of TMD and found an overall incidence of 16%.^[16]^ With such a high prevalence, primary clinical screening for TMD is important. In this study, the right and left lateral height differences between the condyle and mandibular ramus were measured using panoramic radiography. In comparison with MRI, the accuracy of using the height difference for the clinical screening of TMD can be as high as 91.3%. Combining the results of this study with the clinical presentation of the patient allows for the rapid determination of TMJ status. The results of this study, if generalized, will significantly reduce patient waiting time. It also provides a new modality for patients who cannot undergo MRI because of contraindications. Further, it also enriches the clinical application of panoramic radiography in the diagnosis of TMD.

### 4.2. Age selection

Before data measurements can be made, it is important to define the time boundaries between the cessation of jaw development and degenerative changes. This reduces measurement errors caused by issues with bone growth. The condyle is the normal growth area of the mandible.^[17]^ The center of growth and development is located mainly in the condylar cartilage and the fibrous connective tissue overlying it and can be active until the age of 20 years.^[18]^ The probability of developing degenerative osteoarthropathy in people older than 45 years ranges from 14% to 30%. This change leads to friction between the bony surfaces of the joints, and eventually to the formation of bone spurs at the edges of the joints.^[19]^ Therefore, healthy individuals between the age of 20 to 45 years were chosen for the study to prevent measurement inaccuracies caused by bone development.

### 4.3. Panoramic radiographs related

Panoramic radiography has the advantages of low radiation exposure, and simple and rapid shooting. Therefore, it is widely used in the clinical setting. However, because of the magnification problem, it is generally used only for condylar morphology in TMJ-related studies.^[20,21]^ Panoramic radiography has greatly improved shot stability with the use of standardized head positioning and bite blocks. Correlative studies demonstrated the reproducibility of vertical and angular measurements using panoramic radiographs.^[22]^ Further, vertical mandibular height measurements were reliable.^[23]^ At present, the preferred imaging method for TMD is MRI, which can clearly show the structure and characteristics of the articular disc and condylar process, and can analyze and evaluate the displacement angle of the articular disc.^[24,25]^ However, due to its high cost, low penetration rate, frequent adverse events, and other factors^[26]^ its clinical application is limited. Therefore, it is important to develop a simple and fast primary screening method, which is the original intention of this study.

### 4.4. Limitations

Panoramic radiographs focus on bone tissue imaging and have significant limitations in soft tissue imaging. However, they do not show the state of the soft tissues in or around the joint. Consequently, some patients with early TMD and intra-articular soft tissue disease have not yet experienced significant changes in the intra-articular bone. The conditions of these patients could not be accurately determined using panoramic radiographs. This is a possible bias in the selection of research individuals. The final imaging effect of panoramic radiographs depends on the position of the patient, and a certain degree of motion affects the accuracy of the data measurement. Owning to the limitations of data collection and conditions, the weighting of the mandibular ramus and condylar height differences in the diagnosis of TMD has not yet been derived.

### 4.5. Future research directions

In recent years, AI-assisted diagnostic systems have greatly reduced the work intensity of doctors, improved the efficiency of clinical disease screening, and can judge disease status more efficiently, accurately, and objectively.^[27]^ Panoramic radiographs are widely used in the Department of Stomatology and have a large amount of available image data; however, standardized measurement data are still lacking. Our next research goal is to apply deep learning algorithms based on convolutional neural networks to develop an AI-assisted diagnostic system for TMDs. The potential errors in manual spotting measurements can be addressed through extensive model training. The diagnostic capability of panoramic radiographs was further improved by combining them with AI.

## 5. Conclusion

There was a difference between the height of the right and left lateral condyles and height of the mandibular ramus in a healthy population. In healthy population, the height difference between left and right condyle was 1.09 ± 0.99 mm. The height difference of mandibular ramus in male participants was 1.26 ± 0.85 mm, while that in female participants was 1.19 ± 0.87 mm. It was verified by comparison with MRI that in the initial screening of TMD, panoramic radiographs had a low rate of missed diagnoses; however, the rate of misdiagnosis needs to be reduced. Overall, diagnostic accuracy was high (91.3%). Thus, panoramic radiographs, in combination with the patient clinical presentation, can be used for initial screening of TMD. Furthermore, data from panoramic films can be used to create a substantial database of AI-assisted diagnoses.

## Acknowledgments

The authors thank the field workers and other staff who supported in the data collection.

## Author contributions

**Conceptualization:** Zg Wu.

**Data curation:** Xianbin Meng, Sen Liu.

**Formal analysis:** Xianbin Meng, Zg Wu, Liangying Guo.

**Writing – original draft:** Xianbin Meng.

**Writing – review & editing:** Xianbin Meng, Sen Liu, Zg Wu.
